# The Ethnopharmacological Properties of Green-Engineered Metallic Nanoparticles against Metabolic Disorders

**DOI:** 10.3390/medicina59061022

**Published:** 2023-05-25

**Authors:** Neha Rana, Sandeep Kumar Singh, Najitha A. Banu, Ahmed Hjazi, Emanuel Vamanu, Mahendra P. Singh

**Affiliations:** 1 School of Bioengineering and Biosciences, Lovely Professional University, Delhi-Jalandhar Highway, Phagwara 144411, India; 2 Indian Scientific Education and Technology Foundation, Lucknow 226002, India; sandeeps.bhu@gmail.com; 3 Department of Medical Laboratory Sciences, College of Applied Medical Sciences, Prince Sattam bin Adulaziz University, Al-Kharj 11942, Saudi Arabia; 4 Faculty of Biotechnology, University of Agricultural Sciences and Veterinary Medicine, 011464 Bucharest, Romania; 5 Department of Zoology, DDU Gorakhpur University, Gorakhpur 273009, India; 6 Centre of Genomics and Bioinformatics, DDU Gorakhpur University, Gorakhpur 273009, India

**Keywords:** metabolic disorders, green synthesis, metallic nanoparticles, nano-formulation, plant-based nanomedicine

## Abstract

Metabolic syndrome is a multifaceted pathophysiologic condition that is largely caused by an imbalance between caloric intake and energy expenditure. The pathogenesis of metabolic syndrome is determined by an individual’s genetic/epigenetics and acquired factors. Natural compounds, notably plant extracts, have antioxidant, anti-inflammatory, and insulin-sensitizing properties and are considered to be a viable option for metabolic disorder treatment due to their low risk of side effects. However, the limited solubility, low bioavailability, and instability of these botanicals hinder their performance. These specific limitations have prompted the need for an efficient system that reduces drug degradation and loss, eliminates unwanted side effects, and boosts drug bioavailability, as well as the percentage of the drug deposited in the target areas. The quest for an enhanced (effective) drug delivery system has led to the formation of green-engineered nanoparticles, which has increased the bioavailability, biodistribution, solubility, and stability of plant-based products. The unification of plant extracts and metallic nanoparticles has helped in the development of new therapeutics against metabolic disorders such as obesity, diabetes mellitus, neurodegenerative disorders, non-alcoholic fatty liver, and cancer. The present review outlines the pathophysiology of metabolic diseases and their cures with plant-based nanomedicine.

## 1. Introduction

The 21st century has seen unprecedented progress in medical science, which has lowered the mortality rate and successively increased life expectancy. People’s lifestyles have changed dramatically as a result of globalization and modernization, with greater working hours and less leisure time. Furthermore, the frequent use of electronic devices has made life easier and simpler, resulting in the emergence of several lifestyle diseases, one of which is metabolic syndrome [1]. Metabolic diseases occur when the anabolic or catabolic processes of the body are disrupted, causing the body to produce either surplus or inadequate proportions of the key products pivotal for healthy physiological function [2]. The enormity of metabolic risk factors is interconnected to each other, as metabolic syndrome is a constellation of factors that leads to a plethora of pathological manifestations such as obesity, insulin resistance (IR), type 2 diabetes mellitus (T2DM), hypertension, nonalcoholic fatty liver disease (NAFLD), cancer, cardiovascular disorders, and neurodegenerative diseases [3,4,5]. Metabolic syndrome is a multifaceted condition driven by genetic and environmental interaction [6]. Experimental and epidemiological research has suggested that traditional lifestyle modifications, such as a sedentary lifestyle or an energy-dense diet, as well as genetic predisposition, may contribute to the pathogenesis of metabolic diseases [7]. The focal components of metabolic syndrome are increased blood pressure, insulin resistance, high triglyceride levels, reduced HDL cholesterol levels, a large waist circumference, and intra-abdominal fat accumulation. The human body of the twenty-first century is made up of a variety of non-natural chemical mixtures that interact with our cells and organs continually.

### 1.1. Effect of Endocrine-Disrupting Chemicals

Researchers have investigated the links between the chemicals in our food supply and the risk of metabolic illnesses [8]. A family of chemicals that can interfere with the activity of hormones, especially metabolic hormones, has recently gained attention as an environmental factor. Environmental exposure to man-made chemical contaminants is a factor, particularly those that have been found to interfere with hormone action, which are known as endocrine-disrupting chemicals (EDCs) [9]. The endocrine system regulates weight and metabolism by controlling the target tissues and organs. Hormones and growth factors such as estrogens, androgens, glucocorticoids, insulin, and thyroid hormones influence the pathways that influence the adipocyte population and content, as well as hunger, satiety, and energy balance [10].

### 1.2. Effect of Gut Microbiota

A healthy microbiota prevents the body from developing metabolic syndrome, as the gut microbiome is a significant component of human biology in both health and disease [11,12]. *Akkermansia muciniphila* is a mucin-degrading gut bacterium inhabitant of the mucus layer of the gut endothelium. Everard et al. (2013) [13] fed obese mice prebiotic oligofructose, which enriched their guts with *Akkermansia muciniphila* and subsequently treated metabolic syndrome in the obese mice. The administration of *A. muciniphila* enhanced the intestinal level of their endocannabinoids, which govern inflammation, the gut barrier, and gut peptide release. The authors further reported that using *A. muciniphila* membrane protein helps mice with metabolic syndrome. The use of membrane proteins from *A. muciniphila* has been employed to treat metabolic syndrome in mice. Abdominal obesity and insulin resistance are the primary underlying risk factors for metabolic syndrome.

### 1.3. Several Hypothesized Pathways Underlie the Pathogenesis of Metabolic Syndromes Such as Oxidative Stress, Chronic Inflammation, and Insulin Resistance with Fatty Acid Flux [14,15]

#### 1.3.1. Oxidative Stress

Oxidative stress results in the etiology of many diseases. As far as metabolic disorders are concerned, oxidative stress restricts the amount of insulin secretion from the pancreatic β cells and impairs the glucose absorption in muscle and fat cells [16]. Increased oxidative stress affects the vascular wall cells, which leads to atherosclerosis and hypertension [17]. Oxidative stress induces insulin resistance and limits adipocytes from synthesizing adiponectin [18]. Adiponectin protects against the dispersion of severe diseases linked to metabolic disorders and oxidative stress, such as diabetes, cardiovascular disease, and hypertension [19]. Cabandugama et al. (2017) [20] showed a direct connection between obesity and hypertension, as adipose tissue stimulates the adrenal gland to release mineralocorticoid and acts upon the renin–angiotensin–aldosterone system. Hypertension develops as a result of an increase in sodium retention, vascular tone, and a restriction in norepinephrine absorption. As a result, it demonstrates a connection between the pathophysiology of hypertension and fat.

#### 1.3.2. Chronic Inflammation

**Figure 1:**
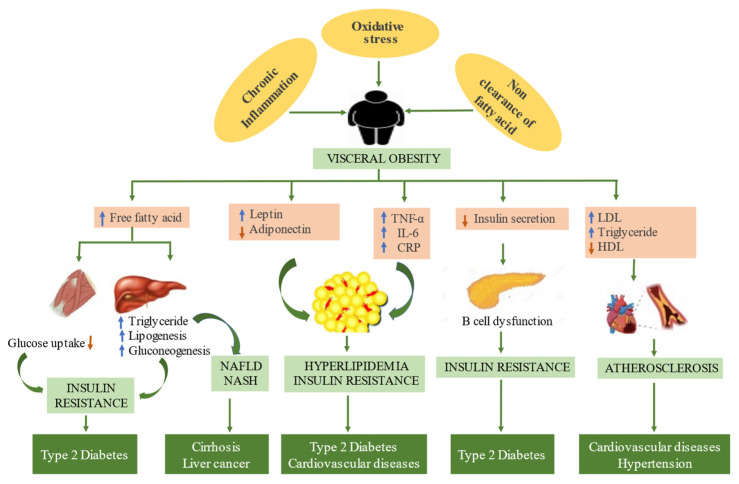
The genesis of metabolic syndrome: chronic inflammation, oxidative stress, and non-clearance of fatty acids are the hallmarks of metabolic syndrome, leading to visceral obesity. Obesity results in elevated levels of free fatty acid, LDL, and triglyceride in serum and liver, consequently causing lipogenesis and gluconeogenesis, leading to insulin resistance. Visceral obesity also results in escalated levels of adipocytokines (leptin, tumor necrosis factor-alpha, interleukin-6, and c-reactive protein) in adipocytes, triggering hyperlipidemia and insulin resistance. Insulin resistance leads to multifaceted metabolic syndrome such as diabetes mellitus, cardiovascular, and hypertension. Cirrhosis and liver cancer are the outcomes of non-alcoholic fatty disease and non-alcoholic steatohepatitis due to ectopic fat accumulation in the liver.

Chronic low-grade inflammation is a significant feature of metabolic diseases. A body’s inability to maintain homeostasis (dysmetabolism) is a crucial factor in low-grade inflammation, eliciting oxidative stress, inflammation, insulin resistance, and a loss of lipid control [21]. Shreds of evidence suggest that there are prominent histological changes in tissues and a phenotypic shift in immune cells that lead to the release of proinflammatory cytokines, proinflammatory lipids, and an array of biological inflammatory mediators, resulting in low-grade inflammation in the systemic inflammatory response [22,23]. Hence, chronic inflammation leads to a loss of homeostasis and further results in various metabolic disorders (Figure 1).

#### 1.3.3. Non-Clearance of Free Fatty Acids

The non-clearance of free fatty acids from the circulation causes insulin resistance in obese individuals. The pancreas secretes a large amount of insulin to overcome this resistance, resulting in hyperinsulinemia [24]. Increased free fatty acid reduces the glucose uptake in muscles; it also induces and suppresses the protein kinase in the liver and muscles, respectively, ultimately leading to an increase in gluconeogenesis [25].

## 2. Plant-Based Nanomedicine

The plant kingdom, known as nature’s pharmacy, is a rich repository of medicinal plants that have been used to cure multiple ailments since ancient times, much before the advent of modern medicine [26]. Numerous natural remedies are proving to be more effective than medications or surgery, without negative side effects. Plants have an orchestra of phytochemical elements, such as polyphenols (flavonoids, phenolic acids), alkaloids, terpenoids minerals, and tannins, which act as secondary metabolites hence, have a critical role the strengthening therapeutic qualities against different ailments. There is a role for polyphenols as direct antioxidants that scavenge ROS, and these ethnobotanicals are in good sync with nature, with no side effects [27]. Achieving a balance between ROS and natural antioxidants is significant and could be a key mechanism for reducing damage related to oxidative stress induced by harmful substances. A study by Hasan et al. (2009) [28] indicated that several chronic ailments, such as diabetes, stroke, gout, asthma, migraine, eczema, fatigue, menopausal symptoms, and irritable bowel syndrome, can be treated with the use of herbal medicines. There are a few constraints, such as the lack of toxicological trials, paucity of the standardization of herbal drugs, low solubility, poor stability, right dosage, short biological half-life, limited oral absorption, and nontarget-specific delivery, which limit the use of herbal drugs at a larger point [29]. These specific limitations have prompted the need for an efficient system that reduces drug degradation and loss, eliminates unwanted side effects and boosts drug bioavailability and the percentage of the drug deposited in the target area. Several drug delivery and drug-targeting systems are currently being developed. There are still requirements for carrier systems that can be useful in herbal drug delivery systems. The quest for an enhanced (effective) drug delivery system has led to the formation of plant-based nanomedicine [30]. This opens up the way for nanomaterials with intriguing physicochemical characteristics, tenability, and versatility to improve disease diagnoses and treatment [31,32].

The advent of nanomedicine in recent years has redefined the dynamics of disease diagnosis and its treatment. Nanomedicine is the clinical application of nanotechnology to improve healthcare and well-being. Nanotechnology is being used in the development of novel medications, leading to the birth of a new era of nanomedicine. The European Union (EU) has designated nanomedicine as a significant Emerging Technology that is capable of delivering novel and innovative medical solutions to address the gaps in healthcare [33,34]. Nanomedicine is the application of nanotechnology to medicine, involving the use of nanoscale materials for disease diagnosis, prevention, control, and treatment [35]. This entails utilizing nanoscale material properties, which might be vastly different from those of the same material on a larger scale [36]. Many biological mechanisms in the human body take place at the nanoscale, and nanoparticles, due to their small size, can penetrate natural barriers and enter new places in the body, interacting with biomolecules in the blood or within organs, tissues, or cells. Nanotechnology is gaining prominence in biology due to its small size, high surface area, increased reactivity, variable pore size, tunable optical, and magnetic properties, particle morphology, and tailored effects [37]. ‘Nano’ is of Greek origin and means “dwarf”, measuring one billionth (10^−9^) of a meter in size [38]. Particles with at least one dimension less than 100 nm are known as nanoparticles and exhibit unique physical and chemical properties, such as optical, thermal, magnetic, and electrical characteristics that make them significant in different sectors in comparison to their larger counterparts [39]. The definition is based on the size of the particles and the proportion of particles that are less than 100 nm in size [40,41]. The nanoparticle is a revolutionary drug carrier for therapeutic and diagnostic purposes, with numerous possible effects such as increasing drug accumulation and bioavailability on the target side, suppressing immunogenicity, and minimizing unwanted effects. Nanoparticles also enhance the solubility of drugs, regulate and maintain drug release, eliminate drugs, and deliver additional drug combinations for a synergistic impact. Nanoparticles can act as “magic bullets”, delivering medications and transferring their contents to specific cellular organelles. Nanoparticles have unique physiochemical properties such as particle shape, size, and hydrophobicity that manifest self-adjuvant effects, hydrophobicity, and release kinetics [42]. The administration of nanoparticles can directly enter the lymphatic system, while larger particles must be internalized by antigen-presenting cells before reaching the lymphatic system, demonstrating the relevance of size in the efficient biodistribution of nanoparticles [43].

Nanoparticles can be fabricated and orchestrated to parcel and carry medications to their destination, shield the drug from premature degradation, keep pharmaceuticals from interacting with the biological environment prematurely, and allow for therapeutic agents to be targeted at the organ, tissue, and cell levels [44]. With this focused strategy, medications do the most damage in the specific and intended location to which they are delivered. This alleviates the collateral harm to the healthy tissues in the surrounding area, as well as side effects [45]. Target-specific delivery overcomes the intrinsic constraints of conventional drugs, such as a short plasma half-life, low stability, the extent of the drug against debasement, and inherent immunogenicity [46]. Nanoparticles are effective drug delivery methods due to their small size. They are beneficial for encapsulating medications and enabling more accurate targeting with sustained release, especially for pharmaceuticals with a low solubility and absorption.

Inorganic NPs have gained interest because of their outstanding properties, such as greater stability, high reactivity, low melting point, and surface plasmon resonance. Their high surface-to-volume ratio makes NPs interact easily with other particles, making diffusion feasible and faster, even at lower temperatures [47,48]. Generally, inorganic nanoparticles are particles that comprise a metal oxide (iron oxide, titanium oxide, zinc oxide, copper oxide, magnesium oxide, and cobalt oxide) or metal (gold, silver, and selenium) central core with a protective organic layer on their surface. The synergistic qualities of the plant and metal NPs are unique in phytonanotherapy because they have therapeutic properties that may be clinically bioequivalent to many chemical-based medications, with minimal adverse effects [36,49]. Engineered nanoparticles, combined with plant-based drug moieties, may be used to ensure an improved efficacy, cellular uptake, specific transport, and delivery of particular molecules because of their modified physicochemical properties.

## 3. Green Synthesis of Nanoparticles

**Figure 2:**
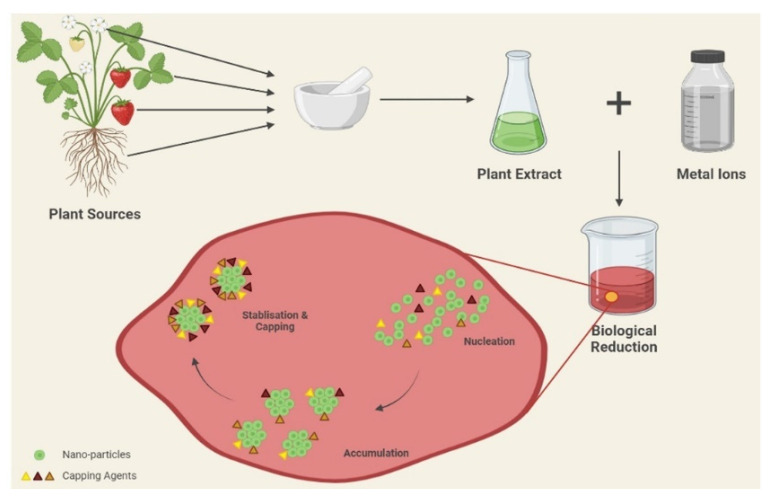
Schematic representation showing the green synthesis of nanoparticles using plant extracts as a remedy for metabolic disorders.

The scientific community has shown great interest in the synthesis of nanoparticles and developed synthetic approaches for the fabrication of these nanoparticles. There are three major ways of synthesizing metallic nanoparticles: chemically, physically, and biologically [50]. The chemical and physical methods are both expensive and foreseeably harmful to the environment, as they involve the use of toxic and hazardous chemicals that are responsible for a variety of biological and environmental risks [51]. Furthermore, physical techniques necessitate a significant amount of energy to maintain the high pressure and temperature required for this synthesis [52]. The usage of hazardous compounds such as reducing agents, organic solvents, and stabilizers has resulted in significant toxicity issues [53]. The chemical synthesis of nanoparticles culminates in some harmful compounds being adsorbed on the surface, which may have negative consequences for medical applications. To combat the issue of toxicity, nanotechnology and green chemistry are combined to create environmentally benign nanoparticles from plants, microorganisms, and other natural resources [54,55]. Green synthesis is considered a clean, reliable, nontoxic, ecologically acceptable, and environmentally favorable approach [56,57,58]. The use of bacteria, cyanobacteria, algae, fungi, and plants has been a witness to the synthesis of metallic nanoparticles [59,60,61,62,63,64,65]. Out of microorganisms, plants have emerged as superior contenders for the biosynthesis of nanoparticles, because microbe-mediated synthesis requires a high maintenance of cultures and aseptic conditions for the growth of microorganisms. Plant-assisted nanoparticle production has a kinetics advantage over other biosynthetic techniques. [66]. Furthermore, there is a usual risk of biohazards with microorganisms. Hence, plant-mediated green synthesis has an edge over other processes. Plant extracts are among the most important bio-reductants, since they are comparatively easy to handle, widely accessible, relatively inexpensive, and have been extensively researched for the green synthesis of other nanomaterials [67,68,69,70,71,72,73,74,75,76] (Table 1). The tautomeric conversion of flavonoids from the enol form to the keto form generates reactive hydrogen species that act as metal ion-reducing agents [77]. Ketones and the carboxylic acid group of flavonoids are also involved in a nanoparticle’s reduction process. The flavonoid Quercetin acts as a chelating agent with metals accountable for the bioreduction of metal ions. In other investigations, other functional groups, such as hydroxyl, carboxyl, amine, cyanide, and ester groups, among others, have been found to work as stabilizing agents for the creation of nanoparticles [78]. Three subsequent phases are involved in the synthesis of metal NPs from plant extracts. During the first phase, the metal ions (M^2+^ or M^+^) get reduced into metal atoms (M^0^), followed by the nucleation of the reduced metal atoms. During the second phase, there occurs the accumulation of small adjacent NPs into larger particles, resulting in enhanced thermodynamic stability. The last phase, the termination phase, provides the final shape of the nanoparticles [79] (Figure 2) The presence of various bioactive molecules as reducing and capping agents without causing toxic effects has attracted much interest in green engineering approaches for nanoparticle synthesis [48].

## 4. Therapeutic Potential of Green Synthesized Nanoparticles

Plants and plant products are proving to be more cost-effective and reliable sources of green synthesis of nanoparticles. Since medicinal plants are renowned for their potential therapeutic applications, the synthesis of nanoparticles using medicinal plants enhances their attributes to a greater extent. Table 2 outlines various types of nanoparticles synthesized from various plant sources via green synthesis, highlighting their therapeutic potential.

### 4.1. Treatment for Cancer

According to emerging research, poor cellular energy metabolism is a defining feature of nearly all cancers, irrespective of their cellular or tissue origin. Chemotherapy is a standard treatment that administers anticancer drugs to patients, limiting malignant cells from proliferating abnormally [80]. The current model of treatment, either oral or parenteral, circulates in the entire body and causes subsequent harm to the normal, healthy, rapidly growing cells in the bone marrow, alimentary canal, macrophages, and hair follicles [81].

Targeted drug therapies utilizing nanosized formulations can effectively address this problem, where only the proliferating cancerous cells are targeted for cytotoxicity. Nanosized formulations are truly a remarkable gift for the treatment of chronic tumors, as conventional cancer therapy and diagnosis have a few disadvantages, including a limited therapeutic efficiency, ineffective targeting, different side effects, and potential biological risks [82,83]. Natural sources have provided approximately 65 percent of the anticancer medicines developed in the last 25 years [84]. The secondary metabolites that have been unearthed in medicinal plant extracts have significant antitumor activity. An excellent active target specificity of plant-based formulations can be achieved by altering the size up to the nanoscale. The efficiency of NPs in the biomedical field largely depends upon the size of a particle, as particles ≥ 100 nm are often more subject to removal from the circulation via phagocytes, while particles ≤ 1–2 nm can simply escape from the normal circulation, causing damage to normal cells and being filtered by the kidneys [85]. NPs with diameters ranging from 10 to 100 nm have significant merits, since they (may) carry medicines efficiently, with an enhanced permeability and retention (EPR) effect, a reduced systemic toxicity, superior pharmacokinetics, and the targeted killing of tumor cells, surpassing drug resistance [86,87].

NPs have a greater tendency to accrue at tumor sites in comparison to healthy tissues, because of the presence of a leaky framework between the endothelial cells and lymphatic drainage. Furthermore, due to insufficient venous and lymphatic clearance, NPs can reside in the tumor microenvironment for a long time [88]. Cancer cells can be distinguished from normal cells by the virtue of the presence of specific ligands on their surface and their irregular shapes. Tumor cells have some distinctive, cell-specific recognition that characterizes them from normal healthy cells at the molecular level. A few receptors, such as the transferrin receptor, folate receptor, and luteinizing hormone-releasing hormone receptor, are over-expressed on their surfaces, offering them a distinctive appearance [89]. The attachment of complementary ligands to the surfaces of these nanoparticles allows them to selectively target malignant cells. Once the nanoparticles attach to the receptors, they are internalized by the cells via receptor-mediated endocytosis or phagocytosis, causing the encapsulated medication to be internalized by the cells [90]. Nanoparticles in cancer cells can amass in the mitochondria, causing a decrease in the mitochondrial membrane potential, thus hampering ATP synthesis and leading to ROS generation [91]. The selective apoptosis of tumor cells and programmed cell death have central roles in cancer treatment. NPs (10–100 nm) entering cancer cells generate ROS, which leads to lipid peroxidation. The mitochondria are targeted and the release of Bax and Bak proteins present on the outer membrane initiates the leakage of cytochrome c (Cyt c). The leakage of Cyt c leads to its binding to caspases, which creates an apoptosome protein complex and ultimately initiates the process of apoptotic signaling in cells [92]. The aforementioned protects normal cells from drug cytotoxicity and aids in the alleviation of the side effects of cancer treatment, which helps to ease the adverse effects of cancer therapy.

**Figure 3:**
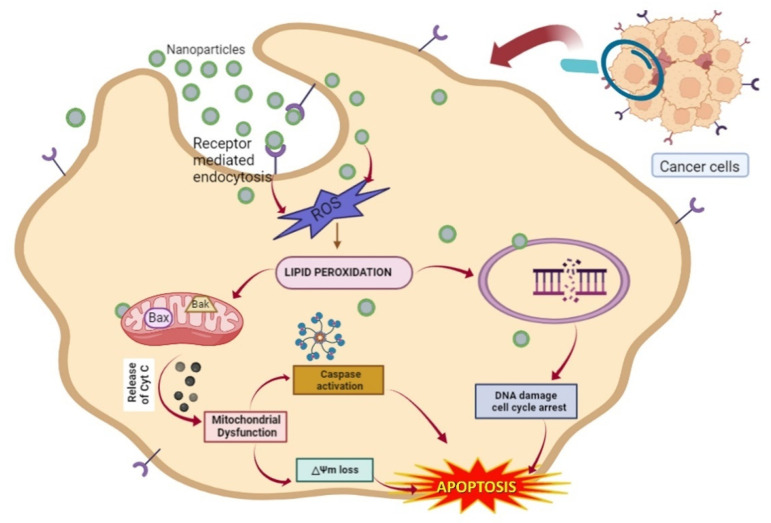
Nanoparticles targeting cancer cells: receptor-mediated endocytosis and phagocytosis lead to the internalization of nanoparticles in the cancer cell. The enhanced permeability and retention (EPR) effect as well as poor lymphatic drainage force nanoparticles to extravasate into tumor masses, and reside in the tumor microenvironment for a long time as compared to normal cells. Upon entering the cancer cell, nanoparticles generate ROS and, subsequently, lipid peroxidation of internal organelles occurs. Bax and Bak proteins on the mitochondrial membrane initiate the leakage of cyt c. There occurs the activation of an apoptosome protein complex by the binding of later to caspases and ultimately initiates the process of apoptotic cell signaling. Additionally, the lipid peroxidation of the nuclear membrane leads to DNA damage, and cell cycle arrest results in the loss of nuclear integrity and finally apoptosis.

Numerous plant-mediated NPs have potent anticancer properties. Kanipandian et al. (2019) [93] utilized silver nanoparticles from *Gossypium hirsutum* to demonstrate the intrinsic apoptotic signaling pathway in A549 lung cancer cells. This has led to the use of NP-based pharmaceuticals that can be widely used in chemotherapy, targeted therapy, radiation, hyperthermia, and gene therapy, as well as in combination therapy [94]. NPs are also supposed to target the nucleus and cause DNA damage, eventually leading to cell cycle arrest [95]. Some cell types experience DNA fragmentation, cell cycle arrest, and apoptosis. A study conducted by Castro-Aceituno and co-workers [96] synthesized Au and Ag NPs from *Plueropetrus multiflorus* (medicinal plant), elucidating their potential against lung cancer. Similarly, *Withania-somnifera*-mediated Se NPs exert growth control against A549 lung carcinoma cells [97]. Studies have demonstrated that *Tamarindus-indica*-mediated silver nanoparticles are cytotoxic to human MCF-7 breast cancer cell lines [98]. The MTT test showed that silver nanoparticles inhibited the proliferation of MCF-7 breast cancer cells in a dose-dependent manner, and their anticancer potential was established using live and dead assays (Ao/EtBr), ROS, and Rho123 assays. The platinum nanoparticles synthesized by using *Nigella sativa* showed remarkable cytotoxic activity against the HeLa cervical and MDA-MB-231 breast cancer line [99]. Green AuNPs derived from *Trachyspermum ammi* seed extract inhibited the proliferation of HepG2 cancer cells in a concentration-dependent fashion, which was associated with ROS-induced death [100]. Recent studies have suggested that apoptosis is caused by plant-based nanoparticles, disrupting the mitochondrial membrane potential as a result of ROS-induced Caspase-3 gene expression and enzyme activity (Figure 3).

### 4.2. Treatment for Diabetes Mellitus

Diabetes is a chronic metabolic disease marked by hyperglycemia that, over time, causes catastrophic damage to the kidneys, heart, eyes, blood vessels, and nerves, and is triggered by a multitude of factors. A sedentary lifestyle, decreased physical activity, obesity, and unavoidable genetic predisposition all contribute to the growing number of diabetes outcomes [101]. Two carbohydrate-digesting enzymes, α-amylase and α-glucosidase, support the digestion of carbohydrates and the absorption of glucose. Restraining the activity of these two enzymes, limiting the digestion of starch and oligosaccharides, subsequently lowering glucose absorption and blood glucose levels as a response aid in slowing the course of diabetes [102].

Several studies have suggested that the leaves of *Ocimum*, rich in phytochemicals such as polyphenols, flavonoids, alkaloids, terpenoids, glycosides, and steroids, can substantially lower fasting and postprandial blood glucose levels [103]. Malapermal and co-workers [104] performed antidiabetic screening on crude extracts of *Ocimum* and *Ocimum*-derived AgNPs. Their results showed the better inhibitory enzymatic activity of carbohydrate-digesting enzymes via Ocimum-derived AgNPs, probably due to the synergistic effect of the various bioactive compounds present in plants and AgNPs. A recent in vitro study by Perumalsamy & Krishnadhas (2022) [105] on *Myristica-fragrans*-mediated silver nanoparticles showed an inhibitory effect on the enzymes α-glucosidase and α-amylase. By adhering to the glucose molecule, the silver nanoparticles effectively restricted the glucose transport across the membrane, establishing antidiabetic activity. Blood glucose concentration is used as a routine and important biochemical measure in the treatment of diabetes to track the disease’s progression. Sengottaiyan et al. (2016) [106] compared the blood glucose levels in diabetes-induced Wistar albino male rats on the 14th and 21st days after the induction of *Solanum nigrum* plant extract and *Solanum-nigrum*-mediated AgNPs. Their results showed the better activity of AgNPs in contrast to the crude plant extract in lowering the blood glucose levels.

*Dittrichia viscosa*, a flowering plant from the Asteraceae family, is known for its hypoglycaemic agents due to the presence of various phytochemicals in the plant [107]. Gold nanoparticles (AuNPs) possess antioxidant and antihyperglycemic effects because of the binding of AuNPs with thioredoxin cysteine residues, which inhibits the inhibitor protein Txnip from latching to them during high glucose levels [108]. The synergistic effect of AuNPs and *Dittrichia viscosa* exhibits wonderful anti-diabetic properties by hindering the absorption of glucose in the intestinal villi and inhibiting the actions of α-amylase and α-glucosidase enzymes by enhancing the secretion of glucagon-like peptide 1 (GLP1) and insulin [109]. Zinc is a crucial micronutrient and zinc deficiency is positively associated with diabetes, owing to the involvement of zinc in insulin production, storage, and secretion, and it being known to improve the structural integrity of insulin [110]. The phytofabrication of Zinc nanoparticles (ZnNPs) using *Vaccinium arctostaphylos* L. fruit extract has an effectual role in in vivo diabetic cures compared to chemically produced ZnO in male Wistar rats [111]. The authors further claimed that bioinspired ZnONPs proved to have better fasting blood sugar levels than their chemically prepared counterparts. Selenium is an important element in the human body, acting as a cofactor and coenzyme of the catalytic-active sites in numerous selenoproteins and enzymes, as well as an antioxidant to safeguard islets of Langerhans from oxidative stress [112]. Selenium nanoparticles (SeNPs) synthesized from aqueous flower extracts of *Cassia auriculata* are reported to exhibit antidiabetic efficacy by inhibiting α-amylase and α-glucosidase activity [113]. In another study, *Pouteria sapota* extract was used in the green synthesis method to fabricate silver nanoparticles, and their antidiabetic activity was tested using in vitro and in vivo models. In vitro, studies confirmed this antidiabetic activity through an observed decline in non-enzymatic glycosylation, the inhibition of -amylase, and an increase in the glucose uptake of yeast cells. However, in streptozotocin-induced rats, the biosynthesized silver nanoparticles markedly increased the activity of superoxide dismutase and catalase, increased the plasma insulin level, and decreased the blood glucose levels [114]. In streptozotocin-induced diabetic rats, the anti-diabetic activity of AuNPs synthesized from *Ziziphus jujuba* was observed. The results demonstrated that biogenic gold nanoparticles are a promising antidiabetic agent [115].

### 4.3. Treatment for Hepatic Injuries

Hepatic metabolism is important in the management of overall energy balance, as the liver plays a central role in the metabolism and detoxification of compounds. Nano-delivery systems enhance the accumulation of drugs in the liver with their bio-filtering activity, which can impede 30–99% of nanoparticles in systemic administration. For nanomaterials aimed at hepatic diseases, this phenomenon becomes an inherent advantage [116].

Curcumin, also known as diferuloylmethane, is a yellow polyphenol derived from the rhizome of the turmeric plant *Curcuma longa*. It has been widely researched for its antifibrotic, antioxidant, anticancer, and anti-inflammatory properties [117]. Pharmacokinetic studies demonstrate the poor water solubility, limited tissue distribution, low bioavailability, and short half-life of curcumin [118]. Highly hydrophobic compounds such as curcumin will most likely benefit from nanoparticle-based delivery methods, which bypass the limitations of poor aqueous solubility. In this light, Adlia et al. (2018) [119] studied the effect of curcumin-fabricated gold nanoparticles on NIH/3T3 cell lines. The authors concluded that the following bio-fabricated nanoparticles demonstrated antifibrotic activity by being non-toxic to NIH/3T3 cell lines. A recent report by Abdullah et al. (2021) [120] pointed out that silymarin-mediated gold nanoparticles (SGNPs) showed a remarkable antifibrotic effect by subduing the activity of the major players (TGFβR1, COL3A1, and TGFβR2) in fibrosis. The resultant effect leads to the stimulation of the transcription factor Nrf2, which is the chief regulator of the antioxidant and cellular protective genes in the liver.

Ectopic deposits of fat (steatosis) in the liver, in the absence of significant alcohol consumption, are known as nonalcoholic fatty liver disease (NAFLD) [121,122]. There is no appropriate treatment for NAFLD except lifestyle changes (weight loss and physical activity) and changes in dietary components [123]. A study by Jazayeri-Tehrani et al. (2019) [124] demonstrated the role of nano curcumin in obese patients with NAFLD by ameliorating glucose index lipids and inflammatory processes.

### 4.4. Treatment for Neurodegenerative Disorders

Neurodegeneration is a hallmark of many chronic, incurable disorders, such as Parkinson’s disease (PD), Alzheimer’s disease (AD), spinal muscular atrophy (SMA), multiple sclerosis (MS), and Huntington’s disease (HD), which are quickly increasing in prevalence [125,126]. With the increase in life expectancy, the prevalence of neurodegenerative diseases has increased manifoldly. Several herbal formulations derived from natural ingredients, such as quercetin (QC), resveratrol (RSV), curcumin (Cur), *Ginkgo biloba*, and *Nigella sativa*, have been shown to boost the state of patients with NDs in prior findings [119,127,128]. The existence of the blood–brain barrier (BBB) in the central nervous system (CNS) is the primary impediment to drug delivery, as it limits the availability of therapeutics to the brain [129]. Nanomaterials tend to penetrate the BBB due to their extended circulation half-life and elevated drug-loading capacity, which boosts the passage of drugs into the brain [130]. The overproduction and aggregation of β-amyloid (Aβ) outside neurons and tau proteins inside neurons lead to the onset of Alzheimer’s disease by impairing synaptic communication and impeding the entry of the molecules and nutrients required for neurons [131]. In one study, phytofabricated gold NPs using *Terminalia arjuna* were effective in inhibiting βA fibrillation and destabilizing mature fibrils. Additionally, the cholinesterases (ChE) were significantly inhibited by the AuNPs, which are used as a cure for Alzheimer’s disease [132].

Parkinson’s disease is primarily caused by nerve cell injury, which is linked to a decrease in dopamine levels in the brain, reducing the ability to control body movements and emotions [133,134]. The seed of *Mucuna pruriens* L. showed promising antiparkinsonian activity due to the presence of L-dopa, which is a precursor for dopamine and can effectively cross the BBB [135]. Tests performed on *Mucuna pruriens* fabricated with different dosages of 5, 10, and 15 mg/kg of body weight reported reduced catalepsy symptoms in mice, with the optimal dose being 5 mg/kg of body weight [136]. A study was carried out by Subakanman et al. (2015) [137] to compare the anti-Parkinson’s efficacy of ethanolic extracts of *Hypericum hookerianum* and *Hypericum-hookerianum*-synthesized gold particles in haloperidol-induced parkinsonian mice. A gait analysis, wire hang test (muscular traction test), and rota rod test, along with a dopamine assay and estimation of glutamate, confirmed the significant neuroprotective effect of *H.-hookerianum*-synthesized gold particles in comparison to the ethanolic extract of *H. hookerianum*. The ability of 1-methyl-4-phenyl-1,2,3,6-tetrahydropyridine hydrochloride (MPTP) to effectively cross the BBB has been demonstrated in in vivo studies on Parkinson’s-disease-induced mice models. Xue et al. (2019) [138] fabricated gold nanoparticles using the root extract of *Paeonia mountan* and studied its effect on MPTP-treated mice. The results showed that, in Parkinson’s-disease-induced mice, gold nanoparticles produced from *Paeonia mountan* suppress neuroinflammation and improve motor coordination.

### 4.5. Treatment for Cardiovascular Diseases

The incidence of cardiovascular disease is rising due to the high intake of saturated fat, salt, and sugar in our diets, sedentary lifestyles, the overconsumption of alcohol, smoking, and an increase in cases of obesity. A cocktail of fat, cholesterol, calcium, and other toxins can form plaques, subsequently leading to atherosclerosis or artery hardening. [139]. Curcumin is a phenolic compound that has long been used to treat atherosclerosis. Studies have shown that curcumin nanoparticles have increased their therapeutic efficacy to manifold effects. Curcumin nanoparticles mitigate myocardial damage in acute myocardial infarction (AMI), as evidenced by the ECGs of rats with isoproterenol-induced myocardial infarction. The curcumin nanoparticles changed the myocardial oxidative stress in these rats [140]. These nanoparticles protected rats against isoproterenol-induced myocardial infarction by changing the ECG and oxidative stress in their myocardial tissue. *Syzygium cumini* (*S. cumini*) is a member of the Myrtaceae family known to have antidiabetic, cardioprotective, and gastroprotective qualities [141]. In contrast, *Syzygium-cumini*-mediated silver particles have proved to have an enhanced cardioprotective effect. *Nyctanthus-arbor-tristis*-mediated ZnO nanoparticles exhibit a strong cardioprotective role against myocardial infarction in rats by improving their serum lipid profile through increasing their HDL cholesterol levels and regulating the high levels of triglycerides, LDL-cholesterol, and total cholesterol levels [142]. Recently, Sui et al. (2022) [143] studied the cardioprotective role of green-synthesized iron nanoparticles from *Calendula officinalis* in HCAEC, HAEC, HCAEC, and HCASMC cells. The research claimed that using iron nanoparticles synthesized by the *C. officinalis* leaf would increase the cardiovascular system’s physiological activity. Another study by Dong et al. (2022) [144] documented the fabrication of *Imperata-cylindrica*-mediated gold nanoparticles and their utility in reducing cardiomyoblastic-induced isoproterenol hypertrophy in the rat cell lines, H9c2 and 3T3.

### 4.6. Treatment for Obesity

Obesity is a major problem, since it is a key risk factor for several noncommunicable diseases, including cardiovascular diseases and type 2 diabetes [145]. The natural products found in various plant resources have modulating effects that impact the control of numerous enzymes and genetic factors [146]. The extract of *Smilex glabra* has the potential to be used in health goods because it is high in phenolics and flavonoids. *Smilax glabra* rhizome-mediated gold nanoparticles changed the anti-obesity status in a high-fat diet and streptozotocin-induced obese diabetes in a rat model [147]. *Salacia chinensis* is a herb known to exhibit a variety of pharmacological effects, including anti-ulcer, anti-antidiabetic, anti-obesity, anti-cancer, hepatoprotective, and skin-lightening agents. The findings by Gao et al. (2020) [148] indicated that *Salacia-chinensis*-loaded gold NPs can decrease the obesity in high-fat-diet-treated mice and prevent the risk of liver disease by increasing adiponectin and decreasing leptin, resistin, and adipose index. The activity of gold nanoparticles synthesized from *Dendropanax morbifera* (D-AuNPs) has antiadipogenic effects on both 3T3-L1 and HepG2 cells via the phenolic compounds present in the plant. The synergistic effect of D-AuNPs has the potential to minimize lipid excess in both cell lines [149]. The in vitro efficacy of *Gynostemma-pentaphyllum-mediated* gold nanoparticles was studied in RAW 264.7 cells and 3T3-L1 cells. The results demonstrated antiadipogenic and anti-inflammatory activity by lowering the accumulation of lipids in the 3T3-L1 obese cell lines and reducing the NO production in the RAW 264.7 cells [150]. Kumar et al. (2023) [151] fabricated copper nanoparticles using *Nigella sativa* seed extracts to investigate their anti-obesity potential. The copper nanoparticles, due to hydrolysis, exhibited an enhanced activity to reduce the fat content, which helps to prevent the conversion of dietary lipids into fatty acids.

**Table 2:** Signified recent work done on green-engineered nanoparticles and their pharmacological role.

Nanoparticles	Plant	Particle Size & Morphology	Pharmacological Role	Test Organism	Ref.
Ag	*Gossypium* *hirsutum*	Size: 13–40 nm Shape: Spherical	Anti-cancer	A549 lung cancer cells	[93]
Ag	*Plueropetrus* *multiflorus*	Size: 20.87 nm Shape: Spherical	Anti-cancer	Lung cancer	[96]
Se	*Withania somnifera*	Size: 40–90 nm Shape: Spherical	Anti-cancer	A549 lung carcinoma cells	[97]
Ag	*Tamarindus indica*	Size: 20–52 nm Shape: Spherical	Anti-cancer	MCF7 breast cancer cell lines	[98]
Pt	*Nigella sativa*	Size: 3.47 nm Shape: Spherical	Anti-cancer	MDA-MB-231, HeLa cancer lines	[99]
Au	*Trachyspermum ammi*	Size: 16.63 nm Shape: Spherical & spheroidal	Anti-cancer	HepG2 cancer cells	[100]
Ag	*Ocimum sanctum* *&* *Ocimum basilicum*	Size: 3–25 nm Shape: Spherical	Anti-diabetic	in vitro	[104]
Ag	*Myristica fragrans*	Size: 50–60 nm Shape: Polygonal	Anti-diabetic	in vitro	[105]
Au	*Dittrichia viscosa*	Size: 20 & 50 nm Shape: Spherical	Anti-diabetic	Sprague–Dawley rat	[109]
ZnO	*Vaccinium* *arctostaphylos*	Size: 40 nm Shape: Spherical	Anti-diabetic	Wistar rats	[111]
Au	*Ziziphus jujuba*	Size: 7–27 nm Shape: Spherical	Anti-Diabetic	Sprague Dawley rats	[115]
Au	*Curcumin*	Size: 50 nm Shape: Spherical	Anti-fibrotic	NIH/3T3 cell line	[152]
Au	*Terminalia arjuna*	Size:19 nm Shape: spherical	Anti-Alzheimer	in vitro	[132]
Ag	*Mucuna pruriens*	Size: 36.5 nm Shape: oval & spherical	Anti-Parkinson	Mice	[136]
Au	*Paeonia mountan*	Size: 100 nm Shape: Spherical	Anti-Parkinson	C57BL/6 mice	[138]
ZnO	*Nyctanthus* *arbour tristis*	Size: 50 nm Shape: spherical	Cardioprotective effect	Albino Wistar rats	[142]
Au	*Imperata cylindrica*	Size: 19.01 nm Shape: Spherical	Cardioprotective	H9c2 and 3T3	[144]
Au	*Smilex glabra*	Size: 20 nm in size Shape: Spherical	Anti-obesity	Wistar rats	[147]
Au	*Salacia chinensis*	Size: 20–50 nm Shape: Spherical	Anti-obesity	Albino Wistar rats	[148]
Au	*Dendropanax morbifera*	Size: 10–20 nm Shape: Spherical	Antiadipogenic effect	3T3-L1 and HepG2 cells	[149]
Au	*Gynostemma pentaphyllum*	Size: 20–200 nm Shape: Spherical	Anti-obesity	3T3-L1 cell lines	[150]
Cu	*Nigella sativa*	Size: 98.23 nm Shape: Spherical	Anti-obesity	in vitro	[151]

## 5. Significant Challenges and Future Perspective

The nanomedicine market is burgeoning due to the emergence of novel drug delivery methods and benefits in different healthcare settings, but the enhanced pharmacokinetics of nanoformulations (absorption, distribution, metabolism, and elimination) may have toxic properties by the virtue of their long-time persistence in humans and the environment [153]. The ever-increasing demand for nanoparticles has enhanced the chance of their accumulation in the ecosystem as well and the long-term effect of these nanoparticles is not yet known [154]. In lieu of the aforementioned, green-synthesized NPs are a much safer alternative in many biomedical applications in comparison to chemically synthesized nanoparticles. In this context, Lakshmi et al. (2015) [155] reported that green-synthesized CuO nanoparticles were significantly more stable than chemically synthesized ones, indicating that they have the desired properties for use in biomedical applications. Similarly, the brine shrimp lethality assay performed by Dowlath and coworkers [156] documented that green-synthesized FeONPs showed minimal toxicity as compared to chemically synthesized FeONPs. The environmentally sustainable technique for NP synthesis is gaining acceptance and is predicted to grow at an exponential rate in the coming years; however, their long-term effects on animals and humans, in addition to the accumulation and influence of these NPs in the environment, must be acknowledged in the future. However, more information from animal models and ongoing, as well as upcoming, clinical trials will be needed to determine whether Phyto nanomedicine will live up to the anticipation aroused by its recent favorable findings in the case of metabolic syndrome [157].

## 6. Conclusions

Worldwide medical and research organizations place a greater importance on metabolic syndrome, since it consists of a group of risk factors that seek improved treatment and prevention measures. As demonstrated in this review, Phyto nanomedicine expedites the development and implementation of technologies or materials that converse with the human body at the molecular level with a high degree of specificity, which can be a good approach to combating the pathophysiology of metabolic syndrome. This approach can be converted into targeted cellular and tissue-specific therapeutic interventions with low to no negative effects and maximum therapeutic potential. Green-synthesized NPs offer intriguing strategies for enhancing encapsulated medication, insulating the payload from deterioration, and targeting particular disease regions. Consequently, plant-based nanomedicine has augmented the bioavailability of plant extracts, as well as their effectiveness, stability, and solubility. Nano herbal formulations are “environmentally friendly and cost-effective” alternatives to traditional approaches; however, to adequately preserve the environment, along with expanding their biomedical applications and ensuring their successful commercialization, it is required that the ecotoxicological impact of green nanoparticles on organisms be elucidated thoroughly.

## Figures and Tables

**Table 1:** Representation of chemical structure of bioactive compounds present in plant extracts acting as a reducing and stabilizing agent in nanoparticle synthesis.

S.No.	Bioactive Compound	Molecular Formula and Structure	Source	Therapeutic Potential	Ref.
POLYPHENOLS					
1.	Quercetin	C_15_H_10_O_7_ 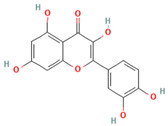	Vegetables, fruits, spices, beverages	Antioxidant Anticancer Anti-diabetic Hepatoprotective Cardioprotective	[67]
2.	Kaempferol	C_15_H_10_O_6_ 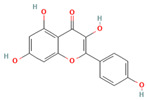	Vegetables, fruits, beverages, medicinal herbs	Antioxidant Anti-cancer Anti-inflammatory	[68]
3.	Rutin	C_27_H_30_O_16_ 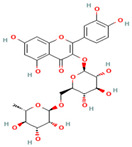	Vegetables, fruits, beverages	Antioxidant Anti-cancer Anti-diabetic Hepatoprotective Neuroprotective	[69]
4.	Myricetin	C_15_H_10_O_8_ 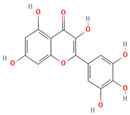	Vegetables, fruits, nuts, berries, herbs	Antioxidant Anti-apoptotic Immunomodulatory Cardioprotective	[70]
5.	Catechin	C_15_H_14_O_6_ 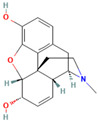	Tea leaves, apricots, broad beans, black grapes, strawberries	Anti-tumor, Anti-inflammatory Anti-bacterial	[71]
6.	Curcumin	C_21_H_20_O_6_ 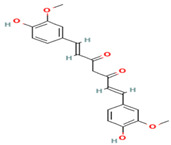	*Curcuma longa*	Anti-inflammatory Analgesic Antipyretic Platelet-inhibitory action	[72]
TERPENOIDS					
1.	Retinol	C_20_H_30_O 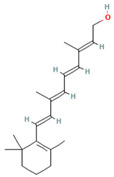	Fruits (mango, papaya) carrot, sweet potato.	Anti-ageing Anti-xerophthalmic Role in embryogenesis	[73]
2.	Menthol	C_10_H_20_O 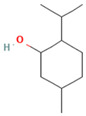	*Mentha arvensis*, *Mentha longifolia*	Local anesthetic agent Antimicrobial Antipruritic	[74]
ALKALOIDS					
1.	Berberine	C_20_H_18_NO_4_^+^ 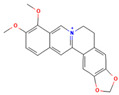	*Coptis chinesis* Genus *Berberis*	Antibiotic, Anti-fungal Antiprotozoal Antidiarrheal Hepatoprotective	[75]
2.	Morphine	C_17_H_19_NO_3_ 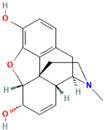	*Papaver somniferum*	Analgesic Antibacterial Neurodegenerative disorders	[76]

## Data Availability

Not applicable.
